# Optimization of Micro-Texturing Process Parameters of TiAlN Coated Cutting Tools by Femtosecond Laser

**DOI:** 10.3390/ma15196519

**Published:** 2022-09-20

**Authors:** Xuefeng Wu, Jinming Zhan, Sanlin Mei

**Affiliations:** Key Laboratory of Advanced Manufacturing and Intelligent Technology, Harbin University of Science and Technology, Harbin 150080, China

**Keywords:** coating tool micro-texture, femtosecond laser, surface structure, tool wear

## Abstract

Coated cemented carbide currently represents most of all cutting tool inserts due to its unique combination of wear resistance and toughness. Surface texturing technology can give additional performance to reduce the tool’s wear and energy consumption. Using TiAlN-coated cemented carbide tools as the research object, the effects of femtosecond laser parameters (laser energy, scanning speed, scanning times) on the groove morphology of TiAlN-coated tools and the bonding state of coating and substrate were discussed. The study found that when the laser energy was 10 μJ, the scanning speed was 0.7 mm/s, and the number of scans was 5, the groove morphology was ideal, and the coating and the substrate combination remained unchanged. The influence of micro-groove texture on the tool wear mechanism was investigated by cutting test using the micro-groove texture tool produced by this group of process parameters. The flank wear value of inserts with micro-grooved texture decreased significantly by around 25%. This work provides practical data to support the femtosecond laser processing of TiAlN-coated tools. It is helpful to understand further the processing mechanism of a femtosecond laser on the micro-texture of coated tools.

## 1. Introduction

Surface micro-texture refers to a kind of surface modification method that uses different machining methods to process micro-structure arrays with specific shapes and sizes on the tool surface to improve the mechanical properties of the tool surface [1,2,3,4]. After surface texture treatment, the formation of the micro-texture array with a corresponding structure and shape on the tool surface has an obvious influence on the lubrication and wear resistance of the tool. It is an effective method to improve the friction characteristics of the material and improve the bearing capacity of the tool surface [5,6,7]. The surface micro-texture with a reasonable structure, width, and layout can effectively reduce the cutting force and tool wear, thereby improving the characteristics of the machined surface formation [8,9,10,11]. The surface microstructure also has great application potential in materials science [12], bionics [13], and medical science [14]. Various coating micro-texture processing technologies have also been developed in the application process of the tool coating micro-texture. The representative technologies include ion etching [15,16], lithography [17,18], EDM [19,20,21], laser processing technology [22,23,24] and so on. Electrical discharge machining (EDM), as a non-contact machining method, can produce various complex and non-standard micro-textures on the tool surface. However, in the machining process, the tool electrode is continuously fed to the workpiece to achieve the discharge distance, so the machining efficiency is low. Lithography is a kind of ultra-fine micro-machining technology with high machining accuracy. However, the slow exposure rate seriously affects machining efficiency, and it is seldom used in machining tool micro-texture. The laser processing technology is relatively convenient in operation, high in processing efficiency, and good in processing quality, so it has been widely used in micro-fabrication.

The femtosecond laser uses self-mode-locking technology to compress the pulse width to the femtosecond level to realize the processing of materials so that the action time of the femtosecond laser and materials is far less than the expansion time of plasma [25]. Therefore, it can avoid the occurrence of the plasma shielding phenomenon and effectively improve the processing efficiency. The femtosecond laser has recently been widely used in tool coating micro-texture processing due to its superior machining characteristics.

Sugihara [26] used a femtosecond laser to process a micro-scale groove texture on the surface of a diamond-like carbon (DLC) coating tool and further studied the influence of micro-texture on different structures and morphologies of tool wear performance. The results show that the micron-level groove texture can significantly improve the adhesive wear resistance of coated tools. The grooved texture prepared by a femtosecond laser on TiAlN-coated tools can reduce the tool–chip contact pressure, store cutting fluid, and reduce chip bonding during cutting [27]. The experiment found that the wear resistance and lubrication ability of TiAlN-coated tools with groove-like micro-textures parallel to the main cutting-edge direction were significantly improved. In addition, Enomoto [28] studied the effect of nano-textures parallel to and perpendicular to the leading cutting edge on the adhesive wear resistance of DLC-coated cutting tools in cutting aluminum alloy. The results show that both textures can improve the adhesive wear resistance of DLC-coated tools and the lubrication ability of the tool surface. Therefore, the tool micro-texture can effectively improve the machining characteristics of the tool material, extending the tool’s life.

After the blade is micro-textured by a femtosecond laser, other methods can be used to improve the cutting performance further. Zhang used femtosecond laser technology to fabricate nanoscale textures on TiAlN-coated tools and used WS2 solid lubricating film to coat nanotextures. The dry cutting performance of the nanoscale textured TiAlN-coated tool deposited with WS2 solid lubricant film is significantly improved, which obtains the smallest cutting force, cutting temperature, and friction coefficient [29]. Currently, most researchers focus on the effect of micro-texture on the cutting performance of tools. However, the effect of femtosecond laser parameters on the quality of the micro-texture and the bonding characteristic between coating and matrix during micro-texture processing is less studied. Improving the processing quality and efficiency without causing processing damage is an essential purpose. The superalloy GH4169 has the properties of fatigue resistance, radiation resistance, oxidation resistance and corrosion resistance. Excellent material properties lead to problems such as a large cutting force, a high cutting temperature, and rapid wear during its processing. The wear of the tool affects the surface quality of the workpiece and reduces the processing efficiency. Therefore, reducing tool wear is an important research direction to solve the machining of superalloys. Micro-textured inserts are micro-structures placed in the tool–chip friction area of the rake face, which can improve the surface lubrication state and the effect of anti-friction and wear reduction. It can effectively reduce friction, reduce cutting temperature, and significantly improve the wear resistance of the insert, thereby enhancing the performance of the cutting tool.

In this paper, the effects of different laser parameters (laser energy, scanning speed, and scanning times) on the micro-structure morphology and the bonding between coating and substrate during the processing of TiAlN-coating tool micro-structure by femtosecond laser are mainly investigated. The processing quality of the femtosecond laser was evaluated by observing the morphology of the grooves. A set of optimal parameters was selected for machining to obtain a micro-structure-coated tool, and cutting experiments were carried out to prove the effectiveness of the micro-texture. This research can provide a practical reference for the parameter selection of TiAlN-coating tool micro-structures in femtosecond laser processing.

## 2. Materials and Methods

### 2.1. Experimental Materials

Coated TiAlN, a physical coating, has strong resistance to chemical attack and heat diffusion and has good results when processing superalloys. Therefore, the TiAlN-coated insert (CNMA120408, KEITO Company, Qingdao, China) is selected as the processing object, and the material of the tool substrate is WC/Co cemented carbide (YG6). The main properties of the tool substrate and coating material are shown in Table 1 and Table 2.

### 2.2. Pretreatment of Materials

**Figure 1:**
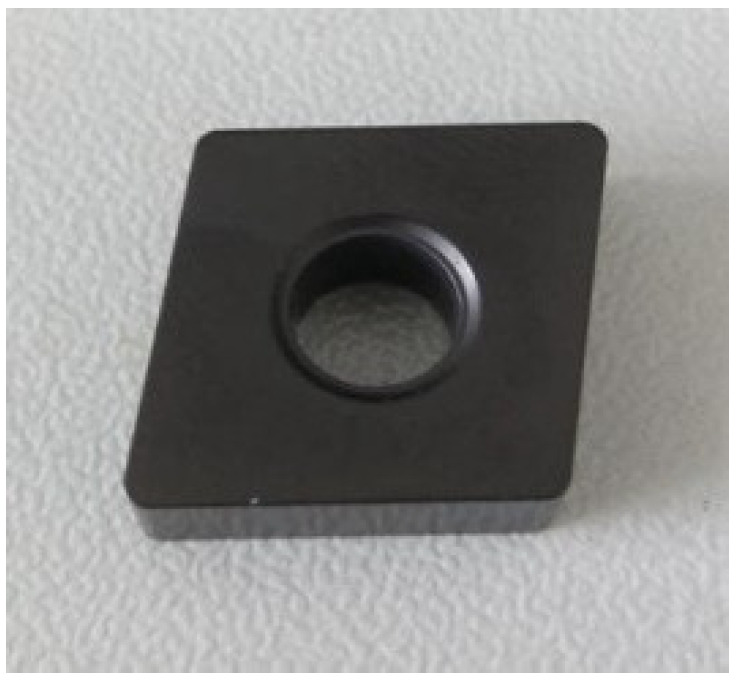
TiAlN-coated.

**Figure 2:**
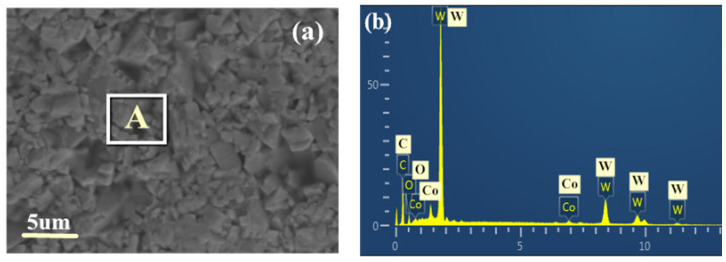

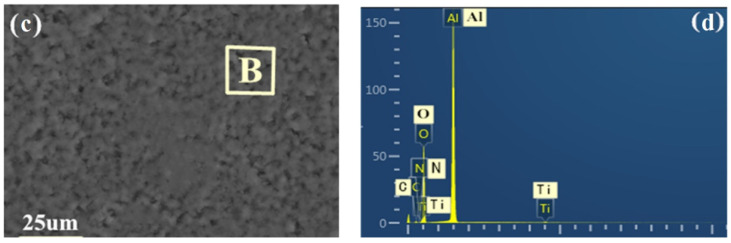
SEM and EDS analysis of the WC/Co matrix and TiAlN coating ((**a**,**b**) are the SEM and EDS of the WC/Co matrix; (**c**,**d**) are the SEM and EDS of TiAlN coating).

Before micro-texturing the substrate surface, the TiAlN-coated tool was first ultrasonically cleaned in acetone and then in alcohol for 30 min (the blade used in the experiment is shown in Figure 1). Figure 2 shows the SEM and EDS diagrams of the TiAlN coating and WC/Co substrate (a and b are the SEM and EDS diagrams of WC/Co substrate, and c and d are the SEM and EDS diagrams of TiAlN coating). In WC/Co cemented carbide, WC has high hardness, Co has good toughness, and the contact angle between WC and Co is zero. Therefore, wettability is excellent, and the liquid phase sintering can be realized at low temperatures [30].

### 2.3. Experimental Methods

**Figure 3:**
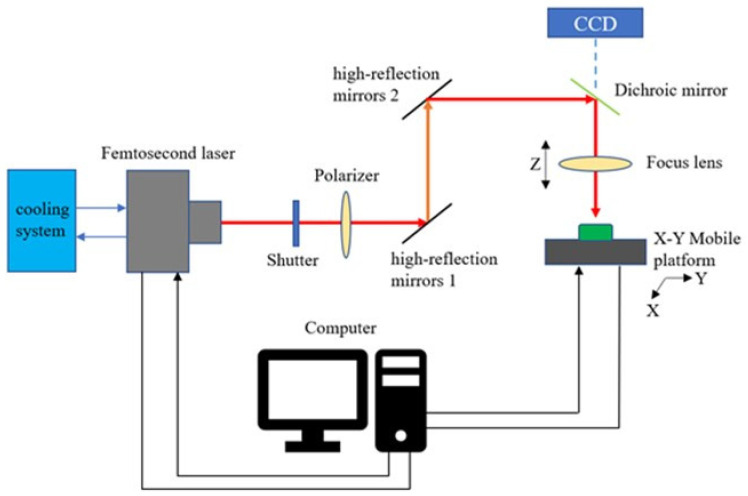
Schematic diagram of femtosecond laser processing system.

The femtosecond laser used in this experiment is a Pharos ultrashort-pulse femtosecond laser (Pharos, Light Conversion Company, Vilnius, Lithuania). The main parameters are shown in Table 3. The laser lens can move up and down in the Z direction. The X-Y mobile working platform is a high-precision motion platform produced by Delong laser (Suzhou, China), and its motion accuracy error is 1 μm. Figure 3 shows the processing system diagram of the femtosecond laser processing TiAlN-coated tool micro-texture, mainly composed of a femtosecond laser generation system, light guide system, workbench, and control system.

In femtosecond laser processing, the workpiece is placed on a high-precision motion working platform, and different laser processing parameters (laser energy, scanning speed, and scanning speed) are selected. The pulse laser is linearly polarized with a high repetition frequency and high energy density is applied to the surface of the processed material. Then it radiates point by point on the sample surface according to the set pattern and equal spacing. Thus, a uniform micro-groove structure with a certain depth and width is machined. After the experiment, the samples were ultrasonically cleaned for 30 min and then dried in cold air. The morphology of the microgrooves machined by a femtosecond laser on the tool substrate surface was observed using a digital scanning electron microscope (SEM, JEOLJSM-6510LV, Hitachi Company, Tokyo, Japan). The chemical composition was analyzed using an X-ray energy dispersive spectrometer (EDS, Oxford INCA Penta FETX3, Oxford Instrument Company, Oxford, UK) equipped with the SEM device.

## 3. Results and Discussion

### 3.1. Analysis of Microstructure Morphology

**Figure 4:**
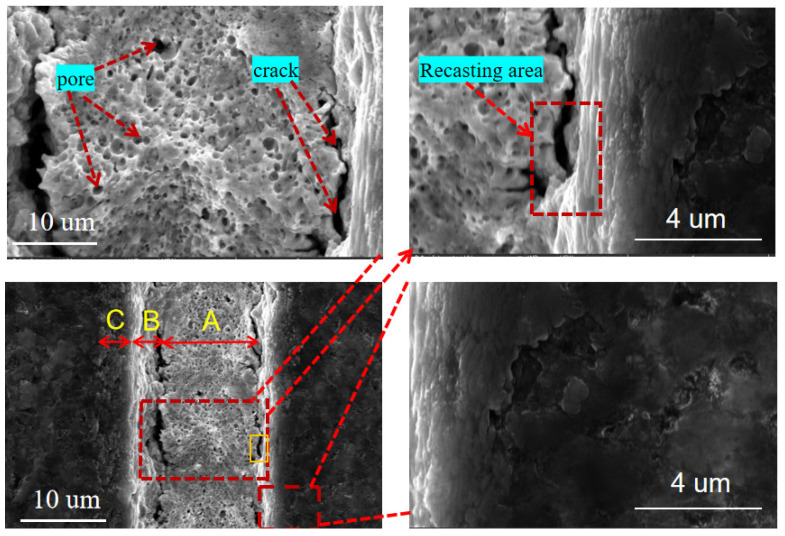
SEM image of microgroove texture on TiAlN-coated tool surface.

Figure 4 shows the micro-texture morphology of the TiAlN-coated tool surface processed by a femtosecond laser when the laser pulse energy is 10 μJ, the scanning speed is 1 mm/s, the number of scans is 3, and the repetition frequency is 40 kHz. The figure that the microgroove texture morphology formed by laser processing can be divided into three different affected areas. The microstructure inside the micro-texture presents a molten structure with a large number of pores, which is a deep ablation area (zone A). Among them, the pores are formed by the explosion of the liquid phase caused by the laser shock [31], as well as the plasma and high-speed gas flow generated by gasification. The molten-like structure is formed by condensing molten droplets that are not sputtered out of the groove in time. When the pressure caused by the laser beam is higher than the surface tension of the tool coating, the melt is sputtered out and condenses in a very short time, creating areas of material recast (zone B) at the edges of the micro-texture. The modified area (zone C) is next to the material recasting area. Although there is no apparent macroscopic morphology change in this area due to the strong Coulomb repulsion, the positive ions ejected after the material cluster explode due to the interrelationship. The formation of larger particles that fail to agglomerate results in the microscopic morphology exhibiting a compact nanoparticle-like morphology.

**Figure 5:**
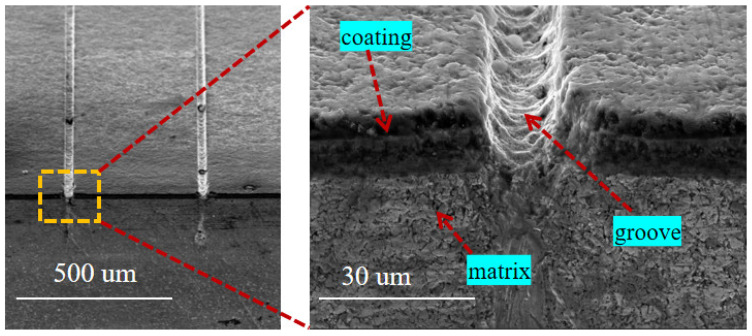
The SEM of the cross-sectional image of the groove structure was processed (laser pulse energy is 10 μJ, the scanning speed is 0.7 mm/s and the scanning number is 5).

**Figure 6:**
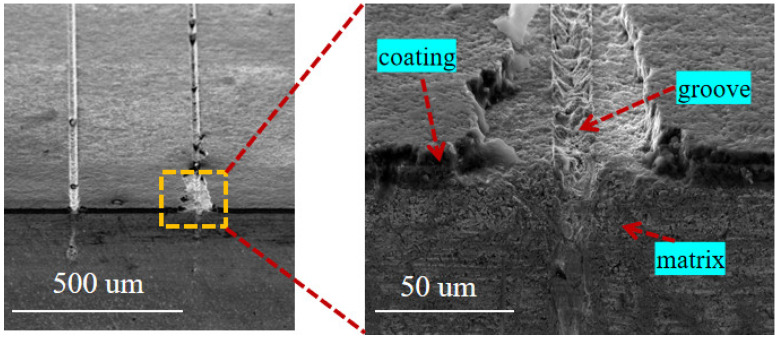
The SEM of the cross-sectional image of the groove structure was processed (the laser pulse energy is 25 μJ, the scanning speed is 2 mm/s, and scanning times is 1).

When the laser pulse energy is 10 μJ, the scanning speed is 0.7 mm/s, the number of scans is 5, and the repetition rate is 40 kHz; the cross-section morphology of the groove structure is shown in Figure 5. It can be seen from the figure that the morphology of the groove is relatively straightforward, there is less molten material in the groove, and there is no significant recast layer produced on the edge of the groove. Moreover, it can be observed that the bonding between the coating and the substrate is good, and the laser at the edge of the groove has caused minor damage to the bonding of the coating and the substrate. Figure 6 shows the microgroove texture morphology when the laser pulse energy is 25 μJ, the scanning speed is 2 mm/s, the scanning time is 1, and the repetition rate is 40 kHz. It can be seen from the figure that due to the high energy of the femtosecond laser and the excessively fast scanning speed, the coatings on both sides of the micro-grooves are severely damaged, and the morphology of the micro-grooves is of poor quality. There is a melting in the micro-grooves, which causes the groove to be blocked.

By comparing Figure 5 and Figure 6, it can be found that when the laser energy is larger, the scanning speed is faster, the scanning times are shorter, the quality of the processed microgrooves is poor, and the damage to the coating is more significant. The resulting stress exceeds the fracture limit of the coating when higher laser energies are used. The machined microgrooves result in a lack of support at the junction of the tool coating and the substrate, providing favorable crack propagation conditions. Finally, the bond between the substrate and the coating is destroyed, and the coating is cracked at the bond. When the scanning method with small energy and multiple times is used for processing, the machined microgroove texture has good morphology, and the laser parameters have little effect on the bonding between the coating and the substrate. Therefore, small energy and multiple scanning times should be used in TiAlN-coated tool micro-texture addition by femtosecond laser.

### 3.2. Experimental Parameters of Laser Processing

The selection of laser processing parameters is essential in the femtosecond laser processing micro-texture process. The width and depth of texture on the machined surface change with the change of laser processing parameters. The femtosecond laser processing system mainly includes laser power, scanning speed, and scanning times. The effects of the above process parameters on the size and micro-morphological characteristics of the micro-trench structure were studied using the single-factor experiment method.

Based on existing research, the processing parameters of a femtosecond laser are screened and optimized, and the actual processing situation is considered. When the output energy of the femtosecond laser is too small, the machined surface cannot form a regular surface texture morphology. When the laser output energy is too large, it will not only affect the morphology of the surface texture but also cause a large number of cracks, pores, and excessive stress concentration on the machined surface. Based on this, the specific range of designed femtosecond laser processing parameters is shown in Table 4.

#### 3.2.1. The Effect of Laser Energy

**Figure 7:**
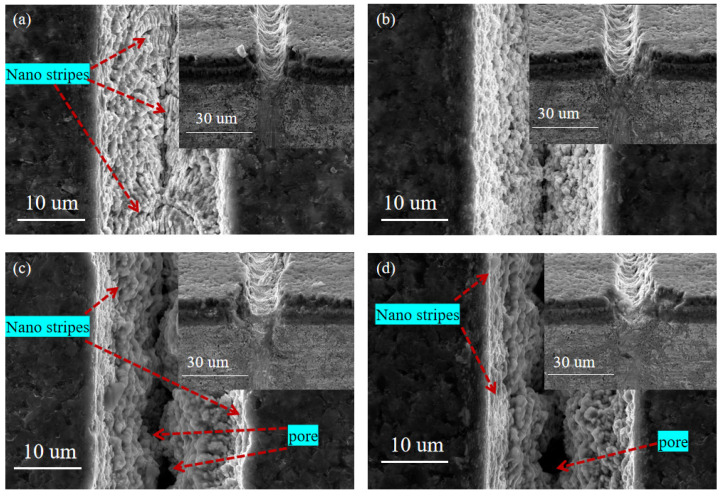
SEM images of micro-texture surface morphology and corresponding section morphology under different laser energy. (**a**) = 5 μJ, (**b**) = 10 μJ, (**c**) = 15 μJ, (**d**) = 20 μJ).

As shown in Figure 7, SEM images of the surface morphology of micro-textures were processed under different laser energy (E) at a scanning speed of 0.7 mm/s, a scanning time of 1, and a repetition rate of 40 kHz. It can be seen from the figure that when the laser energy is small (E = 5 μJ and E = 10 μJ), the microgroove morphology is complete, and the nano-stripe structure can be observed (Figure 7a,b). When the laser energy is increased to 15 μJ and 20 μJ, the energy density at the center of the spot is higher, so the laser energy absorbed by the coating tool material per unit volume is also increased accordingly. The high energy density causes the melting of nano-stripes, and the nano-stripes in the molten state form aggregate under the action of surface tension, which leads to the collapse of the nano-stripe structure at the center of the groove and the formation of large holes. Nano-stripes still exist on both sides of the groove due to low energy (Figure 7c,d). At the same time, the plasma wave caused by high energy density tends to produce a reverse impact, resulting in instability in laser processing and ultimately leading to the unevenness of the machine microgroove edge. Therefore, in the femtosecond laser processing of the TiAlN-coated tool micro-texture, the groove surface morphology is more precise and more regular when the smaller energy (5 μJ or 10 μJ) is selected.

With the increase in laser power, the effective spot diameter of the Gaussian distribution laser spot is higher than the threshold energy density increases, so the shape of the groove is different from the center position of the two sides of the groove. The material absorbs laser energy to produce nonlinear ionization [32] and avalanche ionization [33]. The larger the laser power is, the more obvious the physical process of nonlinear ionization and avalanche ionization is. The deeper the material depth is, the greater the material removal is, and the groove depth increases. The laser energy density is higher than the material combustion threshold with the increased laser power. In conclusion, the power of the femtosecond laser not only affects the groove size obtained by processing and then obtains the micro-textures with different depths and widths, but also has an important impact on the surface quality of the micro-texture.

#### 3.2.2. The Effect of Scanning Speed

**Figure 8:**
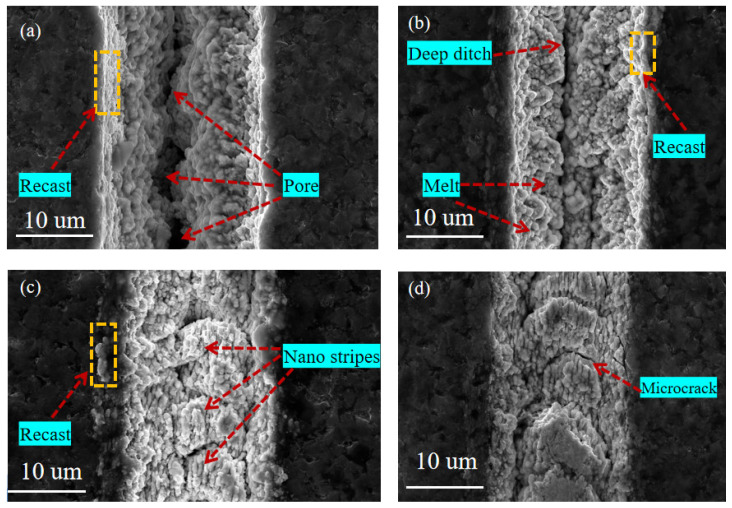
SEM images of micro-texture morphology under different scanning speeds. (**a**) = 0.3 mm/s, (**b**) = 0.7 mm/s, (**c**) = 1.5 mm/s, (**d**) = 2.0 mm/s).

Figure 8 shows the SEM images of the surface morphology of the micro-textures processed at different scanning speeds when the laser energy is 10 μJ, the scanning time is 1, and the repetition rate is 40 kHz.

It can be seen from the figure that when the scanning speed is 0.3 mm/s (Figure 8a), the depth of the groove gradually decreases from the center to both sides, and the edge of the groove is relatively clear. However, the groove has holes in the center and obvious recast layers at the edges. The pores are formed due to the molten droplets, and the internal gas does not escape in time during the condensation process. When the scanning speed is 0.7 mm/s (Figure 8b), the nano-stripes inside the groove collapse and agglomerate to form a melt under the action of surface tension. Deep grooves are formed at the bottom, and recast layers accumulate at the groove edges.

As the scanning speed continues to increase (Figure 8c), the groove depth decreases to a certain extent, and nano-stripes are generated inside the groove. The edge of the groove is blurred, and many melts appear, resulting in uneven edges on both sides. When the scanning speed is 2.0 mm/s (Figure 8d), no obvious groove is formed in the processing area due to the small overlapping area of laser energy. The boundary between the processing and non-processing areas is relatively vague, and there are fine cracks at the edge and inside the groove. These are mainly explosions and plasma caused by high laser radiation energy density causing the formation of high pressure and great thermal shock inside the groove. Based on the above analysis, when the laser scanning speed is 0.3~0.7 mm/s, the micro-texture morphology quality is better, which can meet the actual processing needs.

When other laser parameters remain unchanged, the overlap area of the laser spot is different under different scanning speeds, and the pulse number per unit distance deposited on the surface of the tool material is also different. The two adjacent laser spots have overlapping parts in the laser scanning process. The more overlapping parts of the spots, the more equivalent pulses.

**Figure 9:**
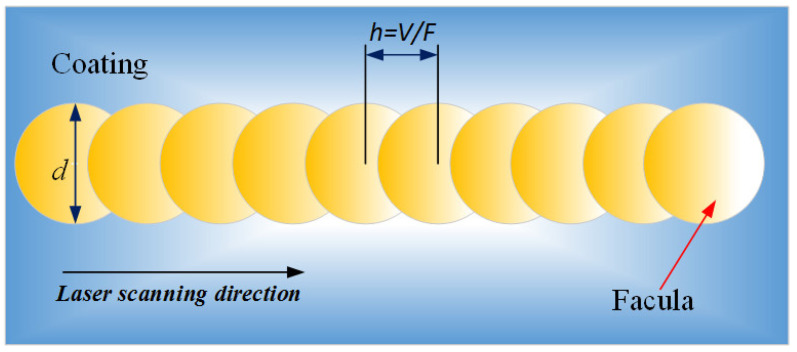
Diagram of laser spot overlap.

**Figure 10:**
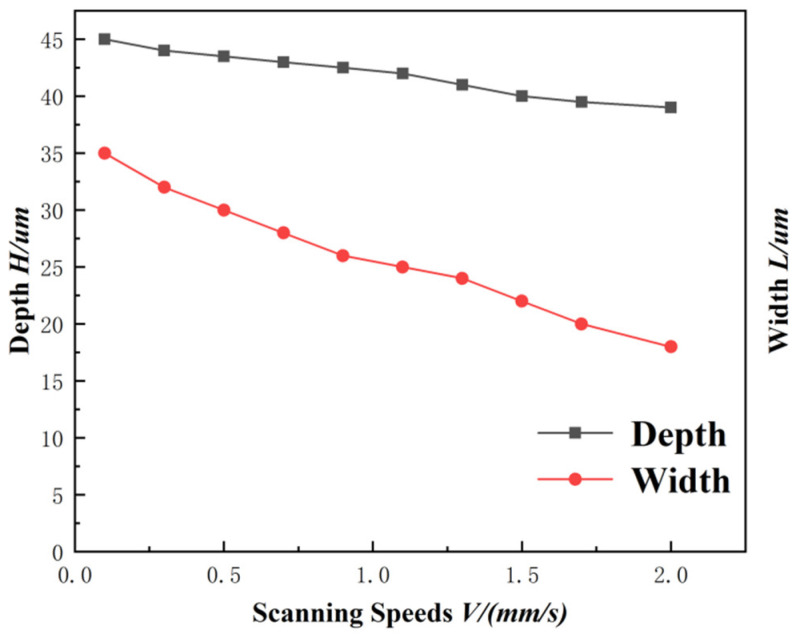
Variation curve of groove depth and width with scanning speed (laser energy is 10 μJ, scanning number is 1, repetition frequency is 40 kHz).

The equivalent pulse number is NV = F/V. When the repetition frequency F of the pulsed laser is constant, the NV is only related to the laser scanning speed. The higher the scanning speed, the lower the laser spot overlap ratio and the fewer laser equivalent pulses (as shown in Figure 9). When the scanning speed is too low, the energy deposited on the unit area is too large, so that the liquid phase in the molten pool cannot be sputtered in time; it thus deposits inside the groove, causing blockage. When the scanning speed increases to a certain extent, the coincidence frequency of the light spot is low, the number of pulses deposited on the unit area is small, and the energy is low, resulting in the shallow depth of the microgroove. The depth and width of the microgroove texture changing with the scanning speed are shown in Figure 10.

#### 3.2.3. The Effect of Scanning Time

**Figure 11:**
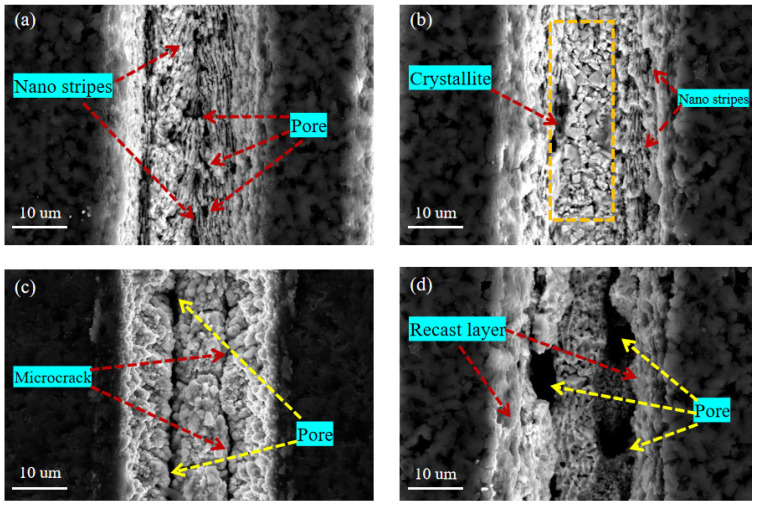
SEM image of micro-texture surface morphology under different scanning times. (**a**) = 1, (**b**) = 3, (**c**) = 5, (**d**) = 8.

Figure 11 shows the SEM images of the micro-texture morphology machines on the surface of the coated tool under different scanning times when the laser energy is 10 μJ, the scanning speed is 0.7 mm/s, and the repetition rate is 40 kHz. It can be seen that when the number of laser scanning is 1 (Figure 11a), the accumulated energy per unit area is insufficient to form apparent groove morphology due to the less absorbed laser energy in the processing area. The groove depth is shallow, the groove boundary is fuzzy, and there are nano stripe structures and pores at the bottom of the groove. When the scanning time increased to 3 (Figure 11b), the microcrystals at the bottom of the groove were observed, the edge of the groove was fuzzy, and the nano-stripe structure was generated.

As the number of scans increases to 5 (Figure 11c), pores and micro-cracks are generated at the bottom of the groove. The edge is relatively straightforward, the molten is less, and the groove morphology is relatively regular. When the number of scans continues to increase to 8 (Figure 11d), there is recast layer deposition inside the groove, resulting in blockage inside the groove. A large number of defects such as pores are distributed inside the groove, and the appearance quality of the groove at the edge is poor. Therefore, excessive scanning times will seriously affect the processing quality of microtextures. When the scanning times are 3~5, the groove morphology is more uniform, and the surface quality is good.

### 3.3. Effect of Micro-Texture on Tool Wear

#### 3.3.1. Micro-Texture Design of the Tool

Micro-textures are usually array structures of micro pits or microgrooves, which are usually arranged in the friction between the tool, the workpiece, and chips. The shape and size of the groove are usually determined according to the processing material, lubrication method, cutting parameters, and other conditions. Grooved-shaped micro-texturing was used for this paper.

**Figure 12:**
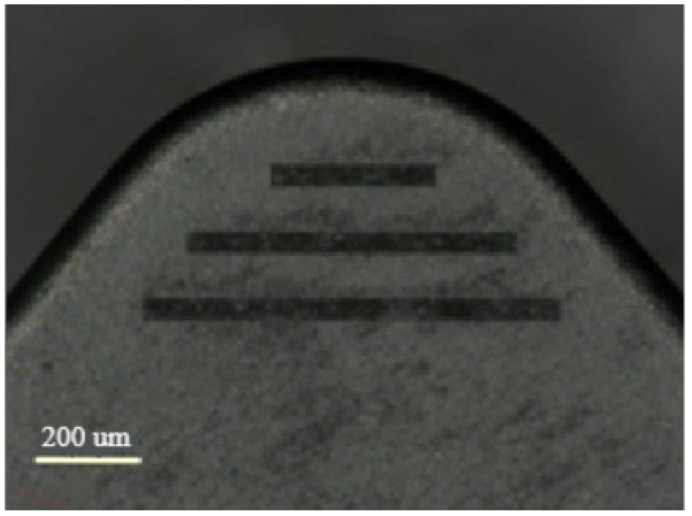
Microgroove texture morphology of rake face.

Since the cutting tool has its specific flow direction in the cutting process, the groove’s direction will affect the chip’s flow and the friction between the chips. According to previous studies, the microgroove texture’s distribution direction directly impacts the tool’s cutting performance. The tool’s cutting performance is better when the angle between the microgroove and the chip’s motion direction is 90° [34]. Therefore, the microgroove texture designed in this paper should be as close as possible to 90° in the direction of the cutting motion (as shown in Figure 12). Moreover, the width of the microgroove texture should be practical and reasonable. If the width of the microgroove texture is too large, the secondary cutting phenomenon will occur, and the cutting force will increase. Therefore, the width size of the microgroove texture should be appropriate.

#### 3.3.2. Trimming Test Analysis

As shown in Figure 12, the microgroove texture is fabricated at 10 μJ laser energy, 0.7 mm/s scanning speed, 5 scanning times, and 40 kHz repetition rate. Table 5 shows the cutting parameters used in the cutting process. Under the cutting parameters, the turning tests were carried out on the non-micro-textured and micro-textured blades, respectively. The wear amount of the two blades was observed by a digital microscope (VHX-1000). The main cutting-edge angle of the blade is 95°, and the rake angle is −6°. The workpiece material is nickel-based superalloy (GH4169).

**Figure 13:**
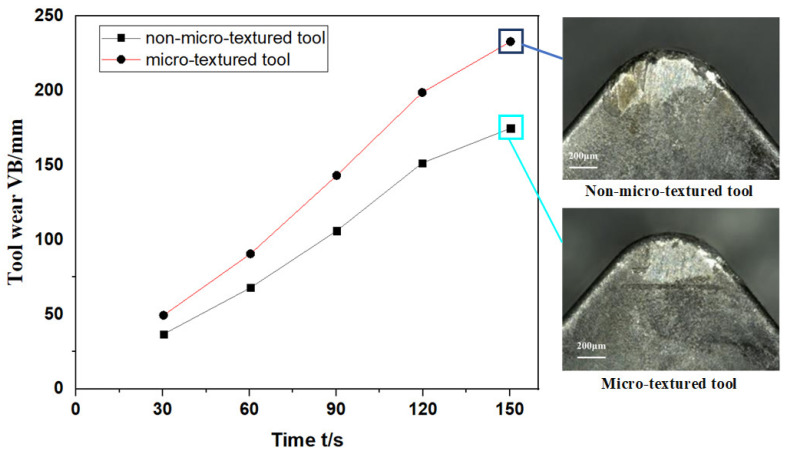
The wear value of different tools varies with time and the adhesion wear image.

The value of flank wear varies with cutting time, and the images of different cutting tools after machining for 150s are shown in Figure 13. The flank wear value of inserts with micro-grooved texture decreased significantly by around 25%. It can be found from the figure that the maximum wear amount of the rake face of the tool with micro-grooved texture is significantly lower than that of the tool without micro-texture, and the wear degree of the top edge of the micro-textured blade is significantly reduced. In the cutting process, the temperature gradient and stress gradient near the contact surface between the tool tip and the workpiece are higher, so there is a significant shear of stress in this area. However, compared with the tool without micro-texture, due to the existence of a micro-grooved texture, the stress concentration of the micro-texture tool tends to the microgroove texture area, which slows down the stress concentration at the edge of the tooltip and plays a role in protecting the tool. In addition, the existence of the micro-texture also reduces the actual contact area between the tool surface and the chip, thereby reducing the average shear strength of the rake face and improving the friction condition of the contact area of the rake face.

**Figure 14:**
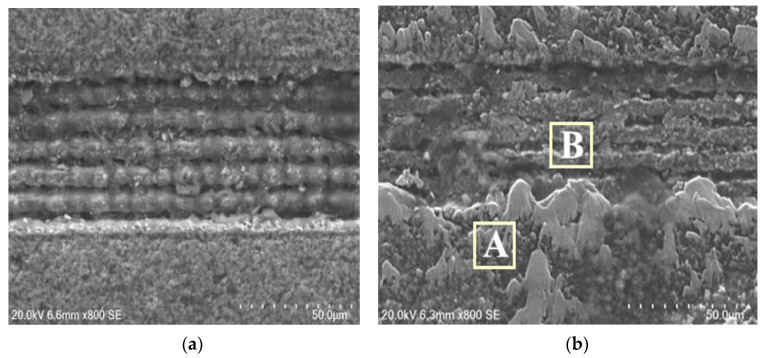
SEM images of microgroove texture before and after cutting. (**a**) Microgroove micro-texture processed by laser, (**b**) Microgroove micro-texture after cutting.

**Figure 15:**
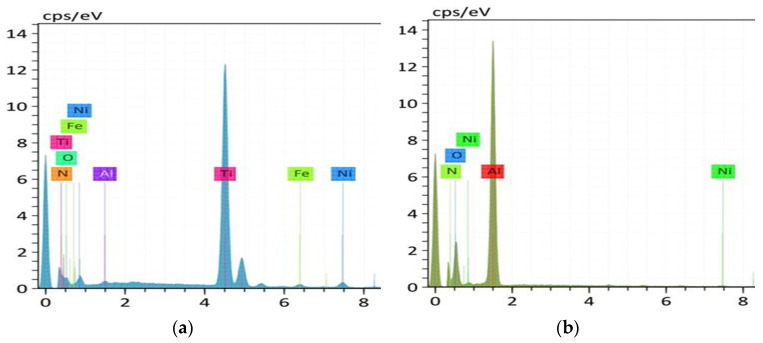
EDS image of the microgroove. (**a**) region on both sides of microgroove, (**b**) inner region with microgroove.

In order to further explore the wear mechanism of the microgroove texture on the tool, an energy dispersive spectrometer (EDS) was used to analyze the specific area. Figure 14 shows the SEM images of the microgroove textured tool before and after cutting, and Figure 15 shows the energy spectrum analysis of the areas on both sides of the microgroove (A) and the internal area of the microgroove (B). It can be seen from Figure 14 that adhesive wear is another factor that influences tool wear. The adhesion on the tool surface can be observed from Figure 14b, and the adhesion in the inner area of the groove is smaller than that in the areas on both sides of the groove. The nickel content in the area without microgrooves is significantly higher than in the areas with microgrooves, indicating that the microgrooves can effectively reduce the bond wear of the tool.

It can be seen that machining microgroove textures on TiAlN-coated tools can effectively reduce the wear of coated tools and improve the tool’s life. However, reasonable femtosecond laser processing parameters and the distribution of microgroove textures are crucial to the tool’s life. Poor microgroove texture morphology cannot improve the tool’s life and also aggravates the tool’s wear.

Although there are many tools for machining superalloys, only TiAlN-coated tools were studied for this paper. Although the processing ability of the femtosecond laser for TiAlN-coated tools is demonstrated, the processing efficiency of the femtosecond laser affects the preparation of micro-textures, and only preliminary experimental studies were carried out. Further research is needed on the type and structure of micro-textures and their processing parameters.

## 4. Conclusions

In summary, we demonstrate the influence of different laser parameters of femtosecond lasers on the machining of TiAlN-coated tools. Under the ultrashort pulse of a femtosecond laser, the small local areas on the TiAlN-coated tool surface undergo drastic changes. The melt and plasma generated by the impact make the micro-texture generated by the processing into three different regions, including a laser burning etched area, a material recast area, and a modified area. After the material absorbs the laser energy, the material is removed by nonlinear ionization and avalanche ionization. Laser energy and scanning speed are the main parameters affecting the micro-texture morphology. Appropriate process parameters can achieve good groove quality and avoid cracks. When the energy of the femtosecond laser is 10 μJ, the scanning speed is 0.7 mm/s, and the number of scans is 5, the quality of the processed microgrooves is the best. Compared to tools without micro-textures, the rake face with micro-textures has a reduced wear value and bond wear during machining, thereby improving tool life.

In the future, more quantitative results will be carried out in follow-up studies. Combined with the cutting effect, the optimization of the micro-texture shape and processing parameters will be comprehensively considered to obtain higher micro-texture processing efficiency without causing damage and improving the cutting tool’s life. Tool materials with more coatings and grades will be used to match suitable cutting and further reduce tool wear during machining.

## Figures and Tables

**Table 1:** Properties of the WC/Co cemented carbide materials.

Composition (wt.%)	Density (g/cm^3^)	Hardness (Gpa)	Flexural Strength (MPa)	Fracture Toughness (MPa m^1/2^)	Thermal Conductivity (W/(m K))	Thermal Expansion Coefficient (10^−6^/K)
WC + 6%Co	14.6	16.0	2300.0	14.8	75.4	4.5

**Table 2:** The properties of the TiAlN coatings.

Coating	Coatings Thickness (um)	Hardness (GPa)	Critical Load (N)
TiAlN	3.0 ± 0.5	34.2 ± 1.5	79 ± 2.3

**Table 3:** Main parameters of femtosecond laser.

Pulse Width (fs)	Central Wavelength (nm)	Repetition Frequency(kHz)	Maximum Monopulse Energy( μJ)	Focus Lens Focal Length (mm)	Spot Diameter (μm)
300	1028 ± 5	40	400	60	11

**Table 4:** The range of the femtosecond laser processing parameters.

Repetition Frequency (kHz)	Laser Energy (μJ)	Scanning Speed (mm·s^−1^)	Scanning Times (n)
40	1–20	0.1–2.0	1–8

**Table 5:** Cutting parameters.

Depth of Cut (mm)	Cutting Speed (m/min)	Feed Rate(mm/r)	Cutting Time (s)
0.3	20	0.2	150

## Data Availability

The raw data are available upon reasonable request to the corresponding author.
